# Investigation of potential early Histologic markers of pediatric inflammatory bowel disease

**DOI:** 10.1186/s12876-015-0359-2

**Published:** 2015-10-13

**Authors:** Julie A. Bass, Craig A. Friesen, Amanda D. Deacy, Nancy A. Neilan, Julia M. Bracken, Valentina Shakhnovich, Vivekanand Singh

**Affiliations:** 1 Division of Gastroenterology, Children’s Mercy Hospitals & Clinics, 2401 Gillham Road, Kansas City, MO 64108 USA; 2 Division of Developmental and Behavioral Sciences, Children’s Mercy Hospitals & Clinics, 2401 Gillham Road, Kansas City, MO 64108 USA; 3 Division of Infectious Disease, Children’s Mercy Hospitals & Clinics, 2401 Gillham Road, Kansas City, MO 64108 USA; 4 Division of Clinical Pharmacology, Toxicology, and Therapeutic Innovation, Children’s Mercy Hospitals & Clinics, 2401 Gillham Road, Kansas City, MO 64108 USA; 5 Department of Pathology, Children’s Mercy Hospitals & Clinics, 2401 Gillham Road, Kansas City, MO 64108 USA

**Keywords:** Inflammatory bowel disease, Eosinophils, TNF-α, MMP-9

## Abstract

**Background:**

Early manifestations of pediatric inflammatory bowel disease (IBD) can be relatively nonspecific. Initial mucosal biopsies may not be conclusive, delaying the diagnosis until subsequent biopsies demonstrate typical histologic features of IBD. We hypothesized that certain inflammatory cell types may be utilized as early histologic indicators of IBD in children.

**Methods:**

A retrospective analysis compared histologic findings from initially inconclusive or negative endoscopic studies in 22 patients who were subsequently diagnosed with IBD (after diagnostic endoscopy) to those of 20 comparison patients with functional abdominal pain matched for age, gender, and study type. A pediatric pathologist, blinded to study group, reviewed biopsies for histologic abnormalities. Eosinophil densities were obtained from the stomach, duodenum, and rectosigmoid areas. Immunohistochemistry (IHC) staining for tumor necrosis factor-α (TNF-α) and matrix metalloproteinase-9 (MMP-9) was performed on the stomach and rectosigmoid areas.

**Results:**

Gastritis and colonic crypt distortion were present in the IBD group at a greater rate (61 % vs. 22 %, *p* = 0.020; 34 % vs. 4 %, *p* = 0.008, respectively). Peak and mean eosinophil densities in the rectosigmoid area were greater in the IBD group (17.0/hpf vs. 5.0/hpf, *p* = 0.0063; 12.3/hpf vs. 4.2/hpf, *p* = 0.0106, respectively). TNF-α and MMP-9 staining did not reveal any significant differences.

**Conclusions:**

Our data suggests that significantly greater inflammation in the stomach, crypt distortion in the colon, and eosinophilia in the rectosigmoid distinguished the IBD group from the comparison group at the time of the initial endoscopic evaluation.

## Background

Diagnosing pediatric inflammatory bowel disease (IBD) may be challenging at times as a broad spectrum of gastrointestinal and extra-intestinal symptoms may complicate the clinical presentation. When IBD is suspected, endoscopy is performed to examine the mucosa and obtain tissue as gross and histologic findings are the gold standard for diagnosing IBD. Unfortunately, histologic confirmation cannot always be obtained early in the course of the disease, thus delaying the diagnosis and treatment, and ultimately the optimal growth potential and quality of life for these patients.

While the quality of the histopathologic diagnosis relies heavily on the clinician to provide helpful clinical information and multiple biopsy specimens from different sites of the gastrointestinal tract, there are well established pathologic criteria for the diagnosis of IBD [1–7]. The typical histologic features of IBD are those of a chronic active colitis, encompassing chronicity features of crypt architectural distortion and basal plasmacytosis. Disease activity is determined by cryptitis, crypt abscesses, and ulcerations. A variable number of eosinophils may be present [1]. Ulcerative colitis (UC) demonstrates a gradation of activity indices depending on the acuity of disease [2, 3]. The inflammatory activity of UC is confined to the mucosa and submucosa; with extension of disease continuously and proximally from the rectum. Mucus depletion from goblet cells, cryptitis with crypt abscesses, and crypt distortion are findings not unique to UC, but may also be seen in Crohn’s colitis [4]. In Crohn’s disease (CD), the inflammation may involve any portion of the gastrointestinal tract from the mouth to the anus. The inflammatory activity is usually discontinuous with skip lesions and shows a transmural involvement [5]. Granulomas are the hallmark lesion of CD but are found in only 40–60 % of resection specimens and much less frequently (15–36 %) in mucosal samples [6, 7].

Importantly, 8–31 % of adult patients with UC and approximately one third of pediatric patients with UC have presented with absent or atypical findings on initial biopsies [2, 8, 9]. There is relative paucity of data addressing this matter specifically in pediatric CD. Studies regarding the diagnostic lag in children with IBD are also lacking. Heikenen et al. retrospectively evaluated 91 children diagnosed with IBD, noting that the average lag for diagnosis was 7.1 months in CD and 6.7 months in UC [10]. Children who presented with growth failure had the longest diagnostic lag. A recent study by Kappelman et al. noted that healthcare utilization by younger IBD patients was disproportionately increased and costs for IBD patients younger than 20 were significantly higher than those for adults, suggesting that effective-management strategies in this population could yield cost-effective benefit [11, 12].

Several cytokines and matrix proteins have been implicated in the pathogenesis of IBD. Tumor necrosis factor-alpha (TNF-α) immunoreactive cells have been noted in increased frequency in the lamina propria of surgically resected specimens of patients with CD and UC [13]. Matrix metalloproteinase-9 (MMP-9) has been suggested as a mediator of mucosal breakdown in IBD and has been shown to be markedly upregulated in intestinal fistulae specimens of patients with CD [14, 15]. However, expression of either TNF-α or MMP-9 is not routinely assessed in the diagnosis of IBD and has not been studied in the early stages of pediatric IBD.

In our pediatric practice, which includes both a large volume of patients with IBD and patients with functional abdominal pain, we have observed that initial biopsies from children with abdominal pain who are later diagnosed with IBD may reveal normal or only subtle non-diagnostic pathologic changes. We hypothesized that there would be a significant difference in the presence of certain histologic findings and inflammatory cell types apparent on review of initial endoscopic biopsies for a subset of diagnostically delayed pediatric IBD patients in comparison to a group of pediatric abdominal pain patients. We further hypothesized that IHC staining for TNF-α and MMP-9 would be helpful in distinguishing the two groups.

## Methods

### Patients

Twenty-two IBD patients (11 CD, 10 UC, and 1 indeterminate colitis) were identified through a gastroenterology departmental IBD database as subjects who had an initial inconclusive endoscopy performed from January 2002 to December 2008 prior to a later confirmatory diagnosis of IBD. Patients who had a definitive diagnosis of IBD on the initial endoscopy were excluded from the study. Approximately 650 patients were diagnosed with IBD during this time period. The comparison group was comprised of 20 patients with a diagnosis of functional abdominal pain identified through a gastroenterology departmental database matched for age, gender, and study type (EGD, colonoscopy, or both). Patients in this comparison group underwent endoscopy between January 2003 and December 2004 and were followed for a time period of at least five years, having not developed IBD during this time. This study was approved by Children’s Mercy Pediatric Institutional Review Board who waived the need for informed consent due to the retrospective nature of the study and analysis of de-identified data and tissue samples.

### Tissue specimens

Biopsy specimens obtained from 37 esophagogastroduodenoscopies (EGDs) and 31 colonoscopies were studied. All biopsies analyzed were obtained during the initial non-diagnostic endoscopy. The EGD specimens included multiple grasp biopsies of the distal esophagus, gastric antrum, and the duodenum. Colonoscopy specimens were obtained from various areas including terminal ileum, cecum, ascending colon, transverse colon, descending colon, and rectosigmoid. Release of tissue samples was approved by the Institutional Review Board and the Chairman of the Department of Pathology at Children’s Mercy Hospital.

### Histopathological evaluation

Sections from the biopsy specimens which had been formalin-fixed and paraffin-embedded in the usual fashion including staining with hematoxylin and eosin were reviewed by a pediatric pathologist, blinded to diagnostic group. Specimens were evaluated for the presence of gastritis, duodenitis, lymphoid hyperplasia, basal plasmacytosis, eosinophilia, cryptitis, crypt abscess, and crypt distortion.

Mucosal (lamina propria) eosinophils of the stomach, duodenum, and rectosigmoid areas were further quantified by the primary investigator (J.A.B.). Eosinophils were identified by the characteristic prominent eosinophilic cytoplasmic granules and a typical bi-lobed nucleus. Densities were determined by counting eosinophils in what appeared to be the most involved area after scanning the entire specimen. Three consecutive high power fields (each high power field approximately 0.15 square millimeters at x400 magnification) were evaluated with the peak count defined as the highest count of the three fields and the mean count as the average of the three fields.

### Immunohistochemistry

Additional slides were prepared from the gastric antrum and rectosigmoid biopsy specimens for IHC staining for tumor necrosis factor-alpha (TNF-α) and matrix metalloproteinase-9 (MMP-9). Serial 4-μm thick sections were cut from paraffin embedded tissue blocks for IHC staining.

IHC staining for TNF-α was performed on the Bond-MAX automated stainer (Leica Corporation, Melborne, Australia). The sections were deparaffinized followed by heat induced antigen retrieval using citrate buffer for 20 min. Mouse monoclonal anti-human TNF-alpha (clone P/T2:AbCam, Cambridge, MA, USA) was used as the primary antibody and applied at a 1:400 dilution. The Bond Polymer Refine Detection kit (Cat. No DS9800 Vision BioSystems BondTM, Newcastle-upon-Tyne, UK) was used as the detection system and included peroxide block, post primary enhancer, poly-HRP anti-mouse/rabbit IgG, DAB chromogen, and hematoxylin counterstain. The stained sections were evaluated for the presence of TNF-α immunoreactive cells and were graded as negative, focal, or diffuse by a pediatric pathologist. The entire specimen was scanned for reactivity. Focal activity was defined as areas of immunoreactive positive cells confined to 1–2 high power fields. Diffuse activity was defined as greater than 2 high power fields of involvement by the immunoreactive cells.

IHC was performed manually for MMP-9. The sections were deparraffinized and then rehydrated in alcohol to tris-buffered saline. Endogenous peroxidase activity was blocked using 3 % hydrogen peroxide followed by a protein block with 5 % goat serum. Residual biotin and avidin activity were quenched using avidin and biotin block, respectively. Affinity purified polyclonal, mono specific rabbit anti-human MMP-9 (HPA001238; Sigma-Aldrich, St. Louis, MO, USA) was used as the primary antibody and applied at a 1:200 dilution overnight at ~4 °C. Labeled streptavidin-biotin (LSAB) was used for the detection system with diaminobenzidine tetrahydrochloride (DAB) as the chromogen. Sections were counterstained with hematoxylin. The stained sections were evaluated for the presence of MMP-9 immunoreactive cells and graded as negative, focal or diffuse by the primary investigator. The grading scheme was similar to that followed for TNF-α.

### Statistical analysis

Comparisons between groups were made by chi-square and Fisher’s exact test as appropriate for categorical variables. Mann–Whitney *U* test was used to compare continuous variables. A significance level of *p* < 0.05 was established for all statistical comparisons. All calculations were performed using the SPSS software package version 17 (SPSS Inc, Chicago, IL).

## Results

### Patients

Demographic and other patient characteristics are presented in Table 1. The number of biopsies per site is presented in Table 2.

**Table 1:** Demographic and clinical characteristics of study groups

Feature	IBD (*n* = 22)	Comparison (*n* = 20)
Mean age (years)	8.9	9.8
Age range (years)	0–16	0–16
Male/female ratio	0.29	0.33
Diagnosis: Ulcerative Colitis	10	n/a
Crohn’s Disease	11	n/a
Indeterminate Colitis	1	n/a
Mean time from first biopsy to diagnosis (months)	23.2	n/a
Range of time from first biopsy to diagnosis (months)	3–72	n/a

**Table 2:** Number of biopsies by site

Site	Total	IBD	Control
Esophagus	34	17	17
Stomach	36	18	18
Duodenum	37	18	19
Rectosigmoid	29	15	14
Left Colon	5	2	3
Transverse Colon	7	3	4
Right Colon	5	3	2
Cecum	12	8	4
Terminal Ileum	25	14	11

### Histology

The histologic abnormalities of the study groups are shown in Table 3. Gastritis (61 % vs. 22 %, *p* = 0.020) and crypt distortion in total colonic biopsies (34 % vs. 4 %, *p* = 0.008) occurred in the IBD group at a significantly greater rate than observed in the control group. In a sub-analysis of the IBD group, the frequency of gastritis and colonic crypt distortion was not different between the UC and CD patients. Other architectural changes and inflammation in individual areas of the colon, terminal ileum, duodenum, and esophagus did not significantly differ between the IBD and comparison group.

**Table 3:** Histologic abnormalities of study groups

	IBD	Control	*p* value
Gastritis	11/18 (61 %)	4/18 (22 %)	.020
Duodenitis	4/18 (22 %)	2/19 (11 %)	.303
Lymphoid hyperplasia: duodenum	1/18 (6 %)	0/19 (0 %)	.486
Lymphoid hyperplasia: cecum	1/8 (13 %)	0/4 (0 %)	.667
Lymphoid hyperplasia: terminal ileum	3/14 (21 %)	0/11 (0 %)	.158
Basal plasmacytosis: rectosigmoid	2/15 (13 %)	0/14 (0 %)	.259
Basal plasmacytosis: transverse colon	1/3 (33 %)	0/4 (0 %)	.429
Basal plasmacytosis: cecum	1/8 (13 %)	0/4 (0 %)	.667
Cryptitis: colon any area	6/37 (16 %)	3/33 (9 %)	.485
Cryptitis: duodenum	2/18 (11 %)	0/19 (0 %)	.230
Cryptitis: rectosigmoid	3/15 (20 %)	1/13 (8 %)	.356
Cryptitis: left colon	0/2 (0 %)	1/3 (33 %)	.600
Cryptitis: transverse colon	1/3 (33 %)	1/4 (25 %)	.714
Cryptitis: right colon	1/3 (33 %)	0/2 (0 %)	.600
Cryptitis: terminal ileum	1/14 (7 %)	0/11 (0 %)	.560
Crypt abscess: colon any area	2/23 (9 %)	0/18 (0 %)	.495
Crypt abscess: rectosigmoid	2/15 (13 %)	0/14 (0 %)	.259
Crypt abscess: cecum	1/8 (13 %)	0/4 (0 %)	.667
Crypt distortion: colon any area	10/29 (34 %)	1/24 (4 %)	.008
Crypt distortion: rectosigmoid	2/15 (13 %)	0/14 (0 %)	.259
Crypt distortion: transverse colon	1/3 (33 %)	0/4 (0 %)	.429
Crypt distortion: right colon	2/3 (67 %)	0/2 (0 %)	.300
Crypt distortion: cecum	5/8 (63 %)	1/4 (25 %)	.273

### Eosinophil counts

Eosinophil counts of study groups are shown in Table 4. Both peak (17.0/hpf vs. 5.0/hpf, *p* = 0.0063) and mean (12.3/hpf vs. 4.2/hpf, *p* = 0.0106) eosinophil densities in the rectosigmoid colon were significantly greater in the IBD group as compared to controls. Gastric and duodenal eosinophil densities did not differ significantly between the two groups. In a sub-analysis of the IBD group, rectosigmoid eosinophil densities did not differ between CD and UC patients. Eosinophil densities were ≥ 20/hpf in only 4 patients and all 4 subsequently developed IBD.

**Table 4:** Eosinophil counts of study groups

	IBD	Control	*p* value
Rectosigmoid mean	12.3 (3.6, 18.6)	4.2 (1.6, 7.0)	.0106
Rectosigmoid peak	17.0 (7.0, 24.0)	5.0 (3.0, 10.0)	.0063
Stomach mean	2.8 (1.3, 4.6)	1.8 (1.0, 2.6)	.3026
Stomach peak	3.5 (2.0, 7.5)	3.0 (2.0, 4.0)	.2775
Duodenum mean	8.1 (6.6, 17.3)	8.8 (8.0, 11.0)	.9495
Duodenum peak	10.5 (8.5, 20.0)	12.0 (10.0, 15.0)	.8488

### Immunohistochemistry

**Fig. 1:**
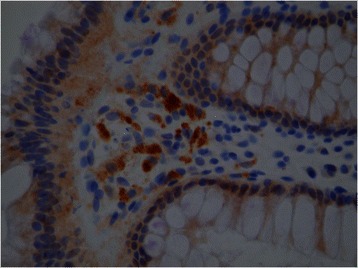
TNF-α. Example of positive TNF-α stain

**Fig. 2:**
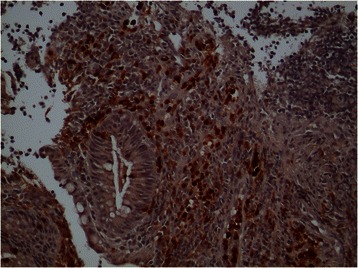
MMP-9. Example of positive MMP-9 stain

IHC staining for TNF-α and MMP-9, Figs. 1 and 2, respectively, did not differ in the stomach or rectosigmoid colon for CD vs. UC or IBD vs. controls. For IBD patients, MMP-9 staining in the stomach was negative in 83 % and focal in 17 %. Gastric MMP-9 was negative in all controls. For IBD patients, MMP staining in the colon was negative in 69 %, focal in 19 %, and diffuse in 13 %. Colonic MMP-9 was negative in 92 % and diffuse in 8 % of controls. For IBD patients, TNF-α staining in the stomach was negative in 89 %, focal in 6 %, and diffuse in 6 %. Colonic TNF-α was negative in 94 % and focal in 6 % of controls. For IBD patients, TNF-α staining in the colon was negative in 87 %, focal in 7 %, and diffuse in 7 %. Colonic TNF-α was negative in 92 % and diffuse in 8 % of controls. The TNF-α immunopositive cells in the mucosa comprised mostly of macrophages and occasionally lymphocytes in the lamina propria.

## Discussion

Our results demonstrated that initial biopsies in children with an eventual diagnosis of IBD in comparison to biopsies of children with functional abdominal pain have significantly greater frequency of inflammation in the stomach, crypt distortion in the colon, and eosinophilia in the rectosigmoid area. This study is novel in that the comparison group is not an adult population but a pediatric subset of abdominal pain patients. Additionally, the IBD group has been expanded to include patients with CD, as prior studies to our knowledge have been limited to findings in UC [16, 17].

This is one of the first studies to attempt to understand the histologic features associated with the development of pediatric IBD. Few studies have addressed the concern that the initial biopsies of IBD patients may be negative or inconclusive and these studies have largely been confined to children with UC [16, 17]. The pediatric IBD population differs from the adult IBD population as children with IBD may present with atypical or normal histologic findings. Markowitz et al. found that 5 of 12 (42 %) children with ulcerative colitis who ultimately required colectomy had rectal sparing or mild patchy inflammation of the rectum and sigmoid areas on initial biopsy [16]. Another study evaluated 73 pediatric patients and 38 adult patients, all with newly diagnosed UC, showing that 30 % of children in comparison with only 3 % of adults had less severe inflammation in the rectum compared with more proximal areas [17]. 21 % of children showed patchy inflammation, which was significantly greater than the adults who had none. Washington et al. demonstrated that chronicity features are lacking more often on initial rectal biopsies in children with ulcerative colitis when compared to adults, finding that the initial biopsies from children were less likely to show diffuse architectural abnormalities when compared to adults [9]. In the current study, we demonstrated that children who will eventually be diagnosed with IBD demonstrate crypt distortion early in their course more frequently than children with abdominal pain not associated with evolving IBD. The presence of crypt distortion should heighten the clinician’s suspicion of IBD even in non-diagnostic biopsies.

Upper endoscopy is considered an essential component of the initial evaluation of a child with possible IBD [18, 19]. In a prospective evaluation of the importance of EGD in the diagnosis of IBD, focally enhanced gastritis was noted in 11 of 21 cases [19]. Focally enhanced gastritis has been seen more commonly in children with CD than UC and has also been noted to be present in 76 % of adults with CD compared to 0.8 % of controls [20, 21]. An increased association of active gastritis in children with CD has also been noted [22]. The gastritis noted in our patients was not active or “focally enhanced” but more indicative of chronic inflammation with a diffuse increase of lymphoplasmacytic cells in the lamina propria with or without reactive epithelial changes. The frequency of gastritis did not differ between CD and UC patients within the IBD group. As our finding of gastritis is rather nonspecific, it invites further investigation regarding the different types of gastritis noted in early IBD in children.

In the current study, we demonstrated significantly higher eosinophil density in the rectosigmoid area of patients who would eventually be diagnosed with IBD. Eosinophil infiltration can be nonspecific and may be observed in conditions such as allergy (e.g. allergy-associated colitis in adults and allergic proctocolitis in infants), eosinophilic gastroenteritis, eosinophilic colitis, IBD, parasite infection, infectious colitis, neoplasm, celiac disease, and autoimmune diseases [23]. Increased eosinophil density has been reported in active inflammation in both CD and UC and is associated with upregulation of eosinophil chemoattractants, eotaxin and RANTES [24]. CD is associated with enhanced secretion and accumulation of eosinophil cationic protein released with eosinophil degranulation [25]. The diagnostic and prognostic values of tissue eosinophilia in IBD patients, however, remain unclear. Some studies have suggested that tissue eosinophilia in the rectum may be predictive of a more favorable prognosis in UC patients as a paucity of eosinophils has been noted to be associated with an increased risk of eventual colectomy [26, 27]. More recent studies, however, have reported a possible dual role for the eosinophil with noted involvement in tissue destruction and repair in the different stages of UC as eosinophils were actually higher in inactive UC than in the active phase of the disease [28, 29]. Shen et al. found that tissue eosinophilia in ileal pouch mucosa in IBD patients treated with restorative proctocolectomy was found to be more prominent than that in the afferent limb, suggesting that luminal factors in different areas of the bowel may contribute to eosinophil-mediated inflammation [30]. Our finding of eosinophilia in the rectosigmoid area of the patients later diagnosed with IBD in comparison to abdominal pain patients suggests that eosinophilia may be an early indicator of disease progression. It should be noted that we did not control for allergic disease which may have influenced the results. However, previous studies have found no association between colonic eosinophil densities and a history of atopy and no significant seasonal variation [31, 32]. Further investigation in a prospective manner with a larger sample size may be warranted to evaluate for the presence of eosinophils in other areas of the gastrointestinal tract.

Prior studies have shown increased expression of TNF-α and MMP-9 in IBD tissue specimens [13–15]. The TNF-α immunohistochemistry by Murch et al. was performed on frozen tissue samples that were not fixed in formalin or paraffin embedded. We failed to detect a significant difference in expression of either marker between the IBD group and the comparison group of abdominal pain patients. This may be due to limited sample size, timing of disease progression, or technical limitations of IHC staining on mucosal specimens that are formalin-fixed and paraffin-embedded. We believe that IHC on formalin-fixed paraffin embedded tissue is less sensitive than western blotting and real time PCR for detecting cytokine expression, however the latter techniques require fresh or frozen tissue. Future studies evaluating TNF-α and MMP-9 on fresh or frozen tissue utilizing western blotting or real time PCR over the course of disease progression may be helpful in understanding the role of TNF-α and MMP-9 as potential early biomarkers for IBD.

There are several limitations to this study, most importantly, the retrospective nature and limited sample size. The initial biopsies of approximately 30 % of the IBD patients included only upper endoscopy specimens. The addition of colon and terminal ileum biopsies in those patients may have provided more diagnostic clues. A prospective design may be challenging as this patient population is difficult to identify in the early stages of disease. Although IHC staining for TNF-α and MMP-9 did not provide significant results in this study, further investigation into other potential IHC markers may be warranted.

## Conclusions

Although typical changes consistent with IBD are present in most cases, occasionally mucosal biopsies will show subtle or nonspecific changes. The absence of chronic changes in the colorectal mucosa on initial biopsies of some children may delay the diagnosis of IBD and initiation of appropriate medical therapy, thus histopathologic criteria predicting disease progression in pediatric IBD may potentially improve patient prognosis and quality of life. Histopathologic findings of colonic crypt distortion and possibly rectosigmoid eosinophilia may be early features of IBD in children. In such cases, careful observation should ensue and consideration should be given to repeat endoscopy if concerning symptoms continue.

## Abbreviations

CD: Crohn’s disease
EGD: esophagogastroduodenoscopy
IBD: inflammatory bowel disease
IHC: immunohistochemistry
MMP-9: matrix metalloproteinase-9
PCR: polymerase chain reaction
TNF-α: tumor necrosis factor-alpha
UC: ulcerative colitis

## References

[1] Jevon G, Ravikumara M (2010). Endoscopic and histologic findings in pediatric inflammatory bowel disease. Gastroenterol Hepatol.

[2] Seldenrijk CA, Morson BC, Meuwissen SGM, Schipper NW, Lindeman J, Meijer CJLM (1991). Histopathological evaluation of colonic mucosal biopsy specimens in chronic inflammatory bowel disease: diagnostic implications. Gut..

[3] Finkelstein SD, Sasatomi E, Regueiro M (2002). Pathologic features of early inflammatory bowel disease. Gastroenterol Clin N Am.

[4] Nikolaus S, Schreiber S (2007). Diagnostics of inflammatory bowel disease. Gastroenterology.

[5] Wolfson DM, Sachar DB, Cohen A, Goldberg J, Styczynski A, Greenstein AJ (1982). Granulomas do not affect postoperative recurrence rates in Crohn’s disease. Gasteroenterology..

[6] Wolfson DM, Sachar DB, Cohen A, Goldberg J, Styczynski A, Greenstein AJ (1982). Granulomas do not affect postoperative recurrence rates in Crohn’s disease. Gasteroenterology..

[7] Keller KM, Bender SW, Kirchmann H, Ball F, Schmitz-Moormann SW, Baumann W (1990). Diagnostic significance of epithelioid granulmoas in Crohn’s disease in children. J Pediatr Gastroenterol Nutr..

[8] Surawicz CM, Haggit RC, Husseman M, McFarland LV (1994). Mucosal biopsy diagnosis of colitis: acute self-limited colitis and idiopathic inflammatory bowel disease. Gastroenterology..

[9] Washington K, Greenson JK, Montgomery E, Shyr Y, Crissinger KD, Polk DB (2002). Histopathology of ulcerative colitis in initial rectal biopsy in children. Am J Surg Pathol..

[10] Heikenen JB, Werlin SL, Brown CW, Balint JP (1999). Presenting symptoms and diagnostic lag in children with inflammatory bowel disease. Inflamm Bowel Dis..

[11] Kappelman MD, Rifas-Shiman SL, Porter CQ, Ollendorf DA, Sandler RS, Galanko JA (2008). Direct health care costs of Crohn’s disease and ulcerative colitis in US children and adults. Gastroenterology..

[12] Kappelman MD, Porter CQ, Galanko JA, Rifas-Shiman SL, Ollendorf DA, Sandler RS (2011). Utilization of health care resources by U.S. children and adults with inflammatory bowel disease. Inflamm Bowel Dis.

[13] Murch SH, Braegger CP, Walker-Smith JA, MacDonald TT (1993). Location of tumour necrosis factor α by immunohistochemistry in chronic inflammatory bowel disease. Gut..

[14] Baugh MD, Perry MJ, Hollander AP, Davies DR, Cross SS, Lobo AJ (1999). Matrix metalloproteinase levels are elevated in inflammatory bowel disease. Gastroenterology..

[15] Kirkegaard T, Hansen A, Bruun E, Brynskov J (2004). Expression and localization of matrix metalloproteinases and their natural inhibitors in fistulae of patients with Crohn’s disease. Gut..

[16] Markowitz J, Kahn E, Grancher K, Hyams J, Treem W, Daum F (1993). Atypical rectosigmoid histology in children with newly diagnosed ulcerative colitis. Am J Gastroenterol..

[17] Glickman JN, Bousvaros A, Farraye FA, Zholudev A, Friedman S, Wang H (2004). Pediatric patients with untreated ulcerative colitis may present initially with unusual morphologic findings. Am J Surg Pathol..

[18] Lemberg DA, Clarkson CM, Bohane TD, Day AS (2005). Role of esophagogastroduodenoscopy in the initial assessment of children with inflammatory bowel disease. J Gastroenterol Hepatol..

[19] Castellaneta SP, Afzal A, Greenberg M, Deere H, Davies S, Murch SH (2004). Diagnostic role of upper gastrointestinal endoscopy in pediatric inflammatory bowel disease. J Pediatr Gastroenterol Nutr..

[20] Sharif F, McDermott M, Dillon M, Drumm B, Rowland M, Imrie C (2002). Focally enhanced gastritis in children with Crohn’s disease and ulcerative colitis. Am J Gastroenterol..

[21] Oberhuber G, Puspok A, Oesterreicher C, Novacek G, Zauner C, Burghuber M (1997). Focally enhanced gastritis: a frequent type of gastritis in patients with Crohn’s disease. Gastroenterology..

[22] Pascasio JM, Hammond S, Qualman SJ (2003). Recognition of Crohn’s disease on incidental gastric biopsy in childhood. Pediatr Dev Pathol.

[23] Mueller S (2008). Classification of eosinophilic gastrointestinal diseases. Best Pract Res Clin Gastroenterol.

[24] Jeziorska M, Haboubi N, Schofield P, Woolley DE (2001). Distribution and activation of eosinophils in inflammatory bowel disease using an improved immunohistochemical technique. J Pathol.

[25] Winterkamp S, Raithel M, Hahn EG (2000). Secretion and tissue content of eosinophil cationic protein in Crohn’s disease. J Clin Gastroenterol.

[26] Tanaka M, Saito H, Kusumi T, Shimoyama T, Fukuda S (2002). Morita, et al. Biopsy pathology predicts patients with ulcerative colitis subsequently requiring surgery. Scand J Gastroenterol.

[27] Heatley RV, James PD (1978). Eosinophils in the rectal mucosa: a simple method of predicting the outcome of ulcerative proctocolitis?. Gut.

[28] Lampinen M, Backman M, Winqvist O, Rorsman F, Ronnblom A, Sangfelt P (2008). Different regulation of eosinophil activity in Crohn’s disease compared with ulcerative colitis. J Leukoc Biol..

[29] Lampinen M, Ronnblom A, Amin K, Kristjansson G, Rorsman F, Sangfelt P (2005). Eosinophil granulocytes are activated during the remission phase of ulcerative colitis. Gut..

[30] Shen B, Plesec T, Remzi F, Kariv R, Lopez R, Queener E (2008). Evaluation of tissue eosinophilia in the pouch and afferent limb in patients with restorative proctocolectomy. Inflamm Bowel Dis..

[31] Polydorides AD, Banner BF, Hannaway PJ, Yantiss RK (2008). Evaluation of site-specific and seasonal variation in colonic mucosal eosinophils. Human Pathol.

[32] Behjati S, Zilbauer M, Heuschkel R, Phillips A, Salvestrini C, Torrente F (2009). Defining eosinophilic colitis in children: Insights from a retrospective case series. J Pediatr Gastroenterol Hepatol.

